# Pneumococcal Septicæmia Starting from a Uterine Focus

**Published:** 1911-07-01

**Authors:** 


### GYNÆCOLOGY.

### PNEUMOCOCCAL SEPTICÆMIA STARTING FROM A UTERINE FOCUS.

The number of different primary sources to which pneumococcal septicæmia has now been traced is considerable. It may be due, for instance, to pneumococcal otitis media, pneumococcal appendicitis, pneumococcal infection of the throat, or to pneumococcal pneumonia, though it is remarkable how seldom lobar-pneumonia leads to widespread pneumococcal infection outside the thorax, and equally remarkable how pneumonia is a relatively uncommon part of most cases of pneumococcal septicæmia. When there is lobar-pneumonia the only common subsidiary infections to occur are pleurisy, empyema, or pericarditis. When there is pneumococcal septicæmia there may or may not be lobar-pneumonia, but there nearly always are pneumococcal pleurisy, pericarditis, peritonitis, and there may be meningitis, arthritis, and other local manifestations of the generalised infection. It would be surprising if the pelvic organs, especially the uterus or its appendages, were not one source of the primary infection in some of these cases of pneumococcal septicæmia, but up to the present in relatively few cases of the kind has this mode of origin of pneumococcal septicæmia been definitely demonstrated. The following, however, is a case in point:—

The patient was a young married woman aged twenty-three, who had had a normal confinement on October 20. The child was born naturally and throve well. The mother seemed also to be in good health. A loss of blood, regarded as return of menstruation, occurred in November, but there was no corresponding period in December. On January 9 a slight blood-stained vaginal discharge set in, together with abdominal pain, pyrexia and vomiting, and the general abdominal symptoms had become so severe by January 11 that a diagnosis of ruptured ectopic gestation was made. On January 12 the vaginal discharge was reddish brown; the uterus could be felt per vaginam to be displaced to the left but not forwards, and the right side of Douglas' pouch was full and boggy, as though there were an inflammatory mass on this side of the pelvis. Acute appendicitis seemed to be by no means impossible, and as the general abdominal condition with the persistent vomiting and pain was unsatisfactory, laparotomy was performed. General peritonitis was found; there was no inflammation of the vermiform appendix except for that amount of reddening of its peritoneal coat which corresponded with the degree of inflammation of the peritoneum in general; but there was greenish-yellow pus, not ill-smelling, in the right Fallopian tube and this was the cause of the abnormal condition felt per vaginam. The appendages on the right side were removed and the pelvis was drained. Cultivations from the right tube gave a pure growth of pneumococci, and it seemed clear that the case had been one of pneumococcal endometritis leading to pneumococcal salpingitis with secondary spread to the general peritoneum, producing general pneumococcal peritonitis. This view was confirmed by the post-mortem examination. At first the patient seemed as though she would do fairly well, but on January 17 abnormal physical signs, suggesting either pleurisy with effusion or else pneumonic consolidation developed at the bases of the lungs; the pulse rate increased to over 140 per minute, although the temperature was only 100° F.; very severe diarrhœa set in and the patient died on January 18.

At the post-mortem examination the uterus was found not to be enlarged and not to present any evidence of recent pregnancy, ectopic or otherwise. Internally, at the lower part of the body and in the cervix, there was a yellowish membranous exudate similar to that which diphtheria produces in the larynx, but proved bacteriologically to be due to pneumococcal endometritis and endocervicitis. There was no evidence of any old-standing pyosalpinx, the purulent infection of the right Fallopian tube that had been dealt with at the time of operation having been quite recent; the left ovary contained an old corpus luteum, and it was quite healthy; the left Fallopian tube was inflamed in the same way that the rest of the peritoneum was, but there was no left-sided pyosalpinx; there was no gastric or duodenal ulcer, no appendicitis, no perforation in the bowel, but the whole of the peritoneal cavity presented a condition of purulent peritonitis, thick greenish-yellow pus and lymph gumming together the greatly distended coils of intestine. There was also bilateral pleurisy verging upon empyema, a pint or more of turbid fluid escaping from both sides of the chest when the thorax was opened, and both lungs being coated with thick buttery lymph. There was no actual lobar-pneumonia, but the lower lobes were rendered partially airless by compression. The pericardium exhibited plastic pericarditis, and it was clear that there had been generalised infection by pneumococci which had first invaded the pelvic organs, thence the peritoneum, and thence again the blood stream.

